# Psychological status of pregnant women during the omicron pandemic outbreak in China

**DOI:** 10.1186/s12905-024-03087-y

**Published:** 2024-06-07

**Authors:** Shuting Bao, Bangwu Chen, Shuqi Zhu, Ying Hu, Chee Shin Lee, Mengkai Du, Menglin Zhou, Danfeng Fan, Biao Xie, Huimin Gu, Zhaoxia Liang

**Affiliations:** 1 Obstetrical Department, School of Medicine, Women’s Hospital, Zhejiang University, Hangzhou, 310006 China; 2 Ninghai Maternal and Child Health Hospital, Ningbo, China

**Keywords:** Omicron, Pregnant women, PHQ-9, GAD-7, ISI, Psychological status

## Abstract

**Background:**

Pregnant women faced great challenges and psychological and physiological changes of varying degrees during the omicron epidemic outbreak. It is important to recognize the potential impact of these challenges on the mental health of pregnant women and to provide appropriate resources and support to mitigate their effects.

**Method:**

By using the convenience sampling approach, a total of 401 pregnant women from two hospitals of different grades in two cities were included in the survey. The cross-sectional survey was conducted by basic characteristics, Generalized Anxiety Disorder (GAD-7), Patient Health Questionnaire (PHQ-9), Insomnia Severity Index (ISI) and self-made questionnaire.

**Results:**

Insomnia affected 207 participants (51.6%), depression affected 160 participants (39.9%) and anxiety affected 151 participants (37.7%). Moreover, pregnant women in provincial capital city were more likely to experience anxiety, depression and insomnia than those in county-level city (*P* < 0.01). Pregnant women’s anxiety, depression and insomnia were positively correlated with the severity of COVID-19 infection (*P* < 0.05). However, COVID-19 infection had no appreciable impact on maternal demand for termination of pregnancy and cesarean section (*P* > 0.05).

**Conclusion:**

Pregnant women frequently suffer from anxiety disorder, depression and insomnia as a result of the omicron pandemic in China. During this period, the community and medical professionals should provide more psychological counseling, conduct health education and offer virtual prenatal care to pregnant women (particularly in the provincial capital city).

**Supplementary Information:**

The online version contains supplementary material available at 10.1186/s12905-024-03087-y.

## Introduction

One of the most catastrophic public health emergencies in the world has emerged since December 2019 due to Coronavirus disease 2019 (COVID-19), which is brought on by the Severe acute respiratory Syndrome Coronavirus 2 (SARS-CoV-2). It has been spreading over the world for three years, and seriously threatens both the physical and emotional wellbeing of people [1]. China has diligently adhered to epidemic control measures during the previous three years. An outbreak of the SARS-CoV-2 Omicron strain took place between December 2022 and January 2023. It is highly contagious, spreads rapidly and widely, poses a major threat to health and life, and induces varying degrees of mental and psychological stress, especially in pregnant women who are already in a special psychological state, and are more prone to adverse psychological changes such as anxiety and depression [2], which have an impact on maternal and child health [3]. During pregnancy, pregnant women experience physiological, psychological and social changes at the same time. During this process, their emotions fluctuate greatly, which is easy to lead to abnormal psychological states such as prenatal anxiety. According to several studies, anxiety, depression, and insomnia are more common during the perinatal period than they are during non-pregnancy periods [4, 5]. Therefore, in addition to thoroughly preventing and controlling the pandemic, it is important to pay attention to the psychological changes that occur in pregnant women and to conduct prompt psychological assessments and effective intervention. In addition, we wanteded to know whether pregnant women infected with Omicron strain have the intention to terminate pregnancy and choose cesarean section, which was not mentioned in the previous literature.

The purpose of this study was to analyze the psychological status of pregnant women and its influencing factors during the omicron outbreak in order to provide sufficient theory and scientific basis for the formulation and implementation of supportive and protective measures aimed at enhancing pregnant women’s mental health.

## Materials and methods

### Recruitment

This study was conducted through an online survey from 10 to 31 January 2023. A total of 401 pregnant women from a hospital in one provincial capital city(Women’s Hospital, School of Medicine, Zhejiang University) (*N* = 191) and a hospital in one county level city(Ninghai Maternal and Child health Hospital (*N* = 210) were included. This study was approved by the hospital’s ethics committee (IRB-20230166-R).

### Questionnaire design

To decrease face-to-face interaction and prevent infection, the invitation was sent to pregnant women via email. People responded to the survey using the online survey tool (Surveystar, Changsha Ranxing Science and Technology, Shanghai, China). Basic characteristics, Generalized Anxiety Disorder (GAD-7), Patient Health Questionnaire (PHQ − 9), Insomnia Severity Index (ISI) and self-made questionnaire make up the five sections of the questionnaire. This questionnaire was used to assess pregnant women’s psychological status between December 8 to December 31, 2022.

1. Basic characteristics: Hospitals, age, level of education and stage of pregnancy were included.
2. Generalized Anxiety Disorder (GAD-7) [6, 7]: The GAD-7 consists of seven items about worry or somatic tension, with response options ranging from 0 to 3 on a 4-point Likert-scale for each item (0 -never; 1 -several days; 2 -more than half the time; and 3 -nearly every day). Anxiety disorders can range in severity from 0 to 21, with higher scores indicating more severe cases. Based on receiver operating characteristics analysis for GAD-7, the cut off points for mild, moderate, and severe anxiety levels are ≥ 5, ≥10 and ≥ 15, respectively.
3. Patient Health Questionnaire (PHQ − 9) [8, 9]: The PHQ-9 questionnaire consists of nine items, and the response options for each item range from 0 to 3 on a 4-point Likert-scale (0 -never; 1 -several days; 2 -more than half the time; and 3 -nearly every day). The total score ranges from 0 to 27, with the higher scores indicating more severe depression. Based on receiver operating characteristics analysis for PHQ-9, the cut off points for mild, moderate, moderate-to-severe, and severe depression are ≥ 5, ≥10, ≥ 15 and ≥ 20, respectively.
4. Insomnia Severity Index (ISI) [10]: A five-point Likert scale is used to rate each item on the ISI, which consists of seven items that evaluate the severity and impact of insomnia (0 = no problem; 4 = very severe problem). The responses are added up to produce a total score, which ranges from 0 to 28. The cutoff points for subthreshold insomnia, moderate insomnia, and severe insomnia, respectively, are ≥ 8, ≥15 and ≥ 22 based on receiver operating characteristics analysis for ISI.
5. Self-made questionnaire: The self-made questionnaire asked questions concerning COVID-19 infection status, delayed prenatal examination, whether family members who live together are infected, desire to terminate a pregnancy, and preference for caesarean delivery. (The questionnaire notes the diagnostic criteria for COVID-19 infection status: Not infected: Nucleic acid test negative; Asymptomatic infection: Nucleic acid test positive, no symptoms; Mild type: The clinical symptoms were mild and there were no signs of pneumonia on imaging; Common type or more serious: There were clinical symptoms with imaging manifestations of pneumonia.)

### Statistical analysis

Scores of several surveys were described as mean ± SD or number (%), along with basic characteristics. The ANOVA test was utilized to compare measurement variables, and followed by Bonferroni’s multiple comparison test. Categorical variables were analyzed by Chi-square test or Fisher’s exact test. If there were non-normal distribution parameters involved, the F test was used. Multivariable logistic regression analysis was performed to identify potential risk factors for participants’ symptoms of anxiety, depression, insomnia, and the desire of terminate a pregnancy. The associations between risk factors and outcomes are presented as odds ratios (ORs) and 95% confidence intervals (CIs), after controlling for confounders. Statistics were deemed significant at *P* < 0.05. The data were analyzed using SPSS software 26.0. Power analysis was conducted by Gpower 3.1 and showed sufficient power(80%) of our study to detect differences in the results.

## Results

### Basic characteristics

The basic characteristics of the participants were shown in Table 1. In the provincial hospital, the average score of GAD-7, PHQ-9 and ISI were 4.97 ± 4.16, 5.60 ± 4.43 and 10.10 ± 4.09. The rate of anxiety disorder, depression and insomnia were 52.4%, 53.9% and 68.6%. Only 15(7.9%) participants were willing to have their pregnancy terminated and 19(12.7%) participants preferred cesarean section. While in the county hospital, the average score of GAD-7, PHQ-9 and ISI were 2.56 ± 3.38, 3.18 ± 3.47 and 7.15 ± 2.87. The rate of anxiety disorder, depression and insomnia were 24.3%, 27.1% and 36.2%. Only 16(7.6%) participants were willing to have their pregnancy terminated and 20(9.5%) participants preferred cesarean section.

**Table 1:** Basic characteristic

Category		mean ± SD/ N (%)	
Hospitals		Provincial hospital	County hospital
Age		31.18 ± 3.81	30.85 ± 4.63
	≤ 29	69 (36.1%)	86(40.9%)
	30–34	93(48.7%)	80(38.1%)
	≥ 35	29(15.2%)	44(21.0%)
Level of education			
	primary school and below	3(1.6%)	8(3.8%)
	Middle school	33(17.3%)	76(36.2%)
	Undergraduate degree	121(63.4%)	123(58.6%)
	Graduate degree	34(17.8%)	3(1.4%)
Stage of pregnancy			
	Early pregnancy	33(17.3%)	60(28.6%)
	Mid pregnancy	55(28.8%)	70(33.3%)
	Late pregnancy	103(53.9%)	80(38.1%)
Anxiety disorder		4.97 ± 4.16	2.56 ± 3.38
	Yes (GAD-7 ≥ 5)	100(52.4%)	51(24.3%)
	No (GAD-7 < 5)	91(47.6%)	159(75.7%)
Depression		5.60 ± 4.43	3.18 ± 3.47
	Yes (PHQ-9 ≥ 5)	103(53.9%)	57(27.1%)
	No (PHQ-9 < 5)	88(46.1%)	153(72.9%)
Insomnia		10.10 ± 4.09	7.15 ± 2.87
	Yes (ISI ≥ 8)	131(68.6%)	76(36.2%)
	No (ISI < 8)	60(31.4%)	134(63.8%)
COVID-19 infection status			
	Not infected	17(8.9%)	19(9.0%)
	Asymptomatic infection	3(1.6%)	12(5.7%)
	Mild type	166(86.9%)	177(84.3%)
	Common type or more serious	5(2.6%)	2(1.0%)
Delayed prenatal examination			
	Yes	61(31.9%)	40(19.0%)
	No	130(68.1%)	170(81.0%)
Whether family members who live together are infected			
	Yes	181(94.8%)	176(83.8%)
	No	10(5.2%)	34(16.2%)
Desire to terminate a pregnancy			
	Yes	15(7.9%)	16(7.6%)
	No	176(92.1%)	194(92.4%)
Preference for caesarean delivery			
	Yes	19(12.7%)	20(9.5%)
	No	131 (87.3%)	190(90.5%)

Provincial hospital: Women’s Hospital, School of Medicine, Zhejiang University; County hospital: Ninghai Maternal and Child health Hospital

### Association between basic characteristics and psychological status

High level hospitals and delayed prenatal examinations were risk factors for anxiety disorders in pregnant women, as shown in Tables 2 and 3. High level hospitals and severe COVID-19 infection status were risk factors for depression. In addition, high level hospital and age were risk factors for insomnia among pregnant women. The difference is statistically significant (*P* < 0.05). There were no significant differences in other categories.

**Table 2:** Association between basic characteristics and psychological status

Category	GAD-7 ≥ 5				PHQ-9 ≥ 5					ISI ≥ 8	
	OR (95%CI)			*P*	OR (95%CI)				*P*	OR (95%CI)	*P*
**Hospitals**											
Provincial hospital	3.426(2.241,5.237)			< 0.001**	3.142(2.072,4.765)				< 0.001**	3.850(2.541,5.832)	< 0.001**
County hospital	Reference			-	Reference				-	Reference	-
**Age**											
≤ 29	Reference			-	Reference				-	Reference	-
30–34	1.390(0.894,2.161)			0.144	1.340(0.868,2.069)				0.186	1.540(1.003,2.365)	0.048
≥ 35	0.962(0.512,1.809)			0.905	0.643(0.336,1.236)				0.182	1.484(0.815,2.701)	0.196
**Level of education**											
primary school and below	Reference			-	Reference				-	Reference	-
Middle school	2.128(0.437,10.375)			0.350	0.696(0.199,2.426)				0.569	1.596(0.442,5.769)	0.475
Undergraduate degree	2.724(0.576,12.883)			0.206	0.792(0.235,2.667)				0.706	2.029(0.579,7.109)	0.269
Graduate degree	6.600(1.246,34.949)			0.026	1.137(0.295,4.388)				0.852	2.059(0.514,8.251)	0.308
**Stage of pregnancy**											
Early pregnancy	Reference			-	Reference				-	Reference	-
Mid pregnancy	1.497(0.846,2.647)			0.166	1.105(0.637,1.918)				0.723	1.182(0.690,2.023)	0.543
Late pregnancy	1.576(0.925,2.684)			0.094	1.151(0.689,1.921)				0.591	1.432(0.868,2.364)	0.160
**Delayed prenatal examination**											
Yes	0.500(1.290,3.225)			< 0.001**	0.587(1.018,2.529)				< 0.001**	1.000(0.823,2.039)	1.000
No	Reference			-	Reference				-	Reference	-
**Whether family members who live together are infected**											
Yes	2.219(1.063,4.635)			0.034*	2.464(1.181,5.142)				0.016*	1.625(0.860,3.069)	0.135
No	Reference			-	Reference				-	Reference	-
**COVID-19 infection status**											
Not infected	Reference			-	Reference				-	Reference	-
Asymptomatic infection	0.143(0.017,1.219)			0.075	0.250(0.028,2.202)				0.212	0.215(0.042,1.099)	0.065
Mild type	1.267(0.613,2.618)			0.523	2.563(1.135,5.787)				0.024	1.639(0.817,3.287)	0.164
Common type or more serious	5.000(0.843,29.656)			0.076	21.000(2.195,200.866)				0.008*	3.500(0.597,20.520)	0.165

Provincial hospital: Women’s Hospital, School of Medicine, Zhejiang University; County hospital: Ninghai Maternal and Child health Hospital

Abbreviations: OR: odds ratio; CI: confidence interval. NA: not applicable; *P < 0.05, ** *P* < 0.001

**Table 3:** Risk factors for physiological status and desire to terminate a pregnancy identified by multivariable logistic regression analysis

Category	GAD-7 ≥ 5	PHQ-9 ≥ 5	ISI ≥ 8	Desire to terminate a pregnancy
	aOR (95%CI) ^a^	aOR (95%CI) ^b^	aOR (95%CI) ^c^	aOR (95%CI) ^d^
**Hospitals**				
Provincial hospital	3.234(2.107,4.966) **	2.978(1.936,4.583) **	3.894(2.556,5.933)**	
County hospital	Reference	Reference	Reference	
**Age**				
≤ 29			Reference	
30–34			1.395(0884,2.200)	
≥ 35			1.756(0.927,3.323)	
**Stage of pregnancy**				
Early pregnancy				Reference
Mid pregnancy				0.261(0.088,0.778)*
Late pregnancy				0.487(0.211,1.122)
**Delayed prenatal examination**				
Yes	2.978(1.087,2.830) **	0.601(0.827,2.166)		0.398(0.184,0.862)*
No	Reference	Reference		Reference
**Whether family members who live together are infected**				
Yes		1.181(0.522,2.671)		
No		Reference		
**COVID-19 infection status**				
Not infected		Reference	Reference	
Asymptomatic infection		0.297(0.032,2.771)	0.279(0.052,1.486)	
Mild type		2.457(1.006,5.996)*	1.702(0.817,3.545)	
Common type or more serious		16.499(1.608,169.303)*	2.625(0.412,16.725)	

Provincial hospital: Women’s Hospital, School of Medicine, Zhejiang University; County hospital: Ninghai Maternal and Child health Hospital

^a^: Adjusted for different hospitals and whether delayed prenatal examination; ^b^: Adjusted for different hospitals, whether delayed prenatal examination, Whether family members who live together are infected and COVID-19 infection status; ^c^: Adjusted for different hospitals, age and COVID-19 infection status; ^d^: Adjusted for pregnancy period and whether delayed prenatal examination

*P < 0.05, ** *P* < 0.001

### Association between basic characteristics and willingness to terminate pregnancy and cesarean section

Mid-pregnancy was a risk factor for pregnancy termination, as demonstrated in Tables 4 and 3, but delaying the prenatal examination was a protective factor. The difference is statistically significant (*P* < 0.05). There was no statistically significant association between basic characteristics and preference for cesarean section. And there were no significant differences in the other categories.

**Table 4:** Association between basic characteristics and desire to terminate a pregnancy

Category	Desire to terminate a pregnancy	
	OR (95%CI)	*P*
**Hospitals**		
Provincial hospital	1.033(0.496,2.152)	0.930
County hospital	Reference	-
**Age**		
≤ 29	Reference	-
30–34	0.749(0.321,1.748)	0.504
≥ 35	1.833(0.710,4.737)	0.211
**Level of education**		
primary school and below	Reference	-
Middle school	0.356 (0.066,1.937)	0.232
Undergraduate degree	0.358(0.072,1.785)	0.210
Graduate degree	0.397(0.057,2.747)	0.349
**Stage of pregnancy**		
Early pregnancy	Reference	-
Mid pregnancy	0.281 (0.095,0.829)	0.021
Late pregnancy	0.559(0.247,1.264)	0.162
**Delayed prenatal examination**		
Yes	0.064(1.091,4.912)	< 0.001**
No	Reference	-
**Whether family members who live together are infected**		
Yes	0.818(0.272,2.458)	0.721
No	Reference	-
**COVID-19 infection status**		
Not infected	Reference	-
Asymptomatic infection	0.786(0.075,8.222)	0.840
Mild type	0.902(0.259,3.142)	0.872
Common type or more serious	1.833(0.162,20.712)	0.624

Provincial hospital: Women’s Hospital, School of Medicine, Zhejiang University; County hospital: Ninghai Maternal and Child health Hospital

Abbreviations: OR: odds ratio; CI: confidence interval. NA: not applicable; *P < 0.05, ** *P* < 0.001

### Correlation between psychological status and COVID-19 infection status

Table 5 shows that compared to women who were not infected or asymptomatic, pregnant women with severe COVID-19 infection status were more likely to suffer from anxiety disorder, depression, and insomnia, the difference is statistically significant (*P* < 0.05). There was no significant difference in desire to terminate a pregnancy.

**Table 5:** Correlation between psychological status and COVID-19 infection status

		COVID-19 infection status				*P*
		Not infected	Asymptomatic infection	Mild type	Common type or more serious	
Anxiety disorder	Yes	12(33.3%)	1(6.7%)	133(38.8%)	5(71.4%)	0.019*
	No	24(66.7%)	14(93.3%)	210(61.2%)	2(28.6%)	
Depression	Yes	8(22.2%)	1(6.7%)	145(42.3%)	6(85.7%)	< 0.001**
	No	28(77.8%)	14(93.3%)	198(57.7%)	1(14.3%)	
Insomnia	Yes	15(41.7%)	2(13.3%)	185(53.9%)	5(71.4%)	0.007**
	No	21(58.3%)	13(86.7%)	158(46.1)	2(28.6%)	
Desire to terminate a pregnancy	Yes	3(8.3%)	1(6.7%)	26(7.6%)	1(14.3%)	0.924
	No	33(91.7%)	14(93.3%)	317(92.4%)	6(85.7%)	

Data were N (%). *P < 0.05, ** *P* < 0.001

## Discussion

Depression and anxiety are the most common mental health problems during pregnancy, with about 12% of women experiencing depression and 13% suffering anxiety at some point. Many women will experience both [4, 11]. During the COVID-19 epidemic, many studies on stress during pregnancy have been carried out, all showing that the psychological burden of pregnant women has increased sharply [12–14]. These findings highlight the possibility that COVID-19 may exacerbate psychological problems in pregnant women, with potential impacts on the developing fetus. Via an online survey, our study looked at pregnant women’s psychological status, demand for abortions and decision to have a cesarean section during the Omicron strain epidemic.

The current study reveals that the psychological health of pregnant women was generally poor during the Omicron strain epidemic, and it was even worse in higher-level hospitals. It has no appreciable impact on pregnant women’s willingness to terminate their pregnancy and to opt for cesarean section, nevertheless.

Similar to our findings, Ayaz R et al. reported that COVID-19 outbreak negatively affects the mental health of pregnant women, which leads to adverse birth outcomes. During the pandemic, pregnant women’s levels of anxiety and depressive symptoms significantly increased [11, 15].

This study found that insomnia was common among pregnant women and positively correlated with age, which is consistent with earlier research [16]. Emotional and sleep disorders are common during pregnancy, and the prevalence of insomnia in pregnant women is significantly higher than in the general population. Epidemiology shows that the incidence of insomnia in the first trimester is approximately 34.00%, and it can reach as high as 68.00–80.00% in the second and third trimesters [17]. Insomnia during pregnancy is often accompanied by emotional disorders such as anxiety and depression. Kizilirmak et al. [18] found that anxiety is more common than depression in pregnant women and is closely related to sleep quality.

Infection with COVID-19 during pregnancy has been linked to severe maternal-fetal, pregnancy-related, and fetal complications, such as miscarriage, intrauterine growth restriction, and preterm birth, as well as increased risk of vertical transmission and maternal mortality [19]. Therefore, pregnant women frequently worry about contaminating themselves, their unborn child, and their elderly relatives during the pandemic [20]. A national retrospective cohort study [21] reported that, when compared to the non-COVID-19 group, women in the COVID-19 group had a higher frequency of cesarean section, although rates of pregnancy terminations at ≥ 22 weeks of gestation were not significantly different. Another study found that during the pandemic,16.3% mothers desired earlier pregnancy termination and 39% requested cesarean Sect. [22]. In our study, 7.7% of pregnant women wanted an early pregnancy termination and 10.8% requested cesarean section, and there was no significant difference between COVID-19 infection status and maternal demand for pregnancy termination and cesarean section. The potential impact of childbirth on the body of the person who gives birth is substantial. Both caesareans and vaginal delivery take time to recover from, the former being surgical and the latter physically exhausting. Further, both may result in scarring. The most common opinion is that caesareans carries greater risks than vaginal delivery and that since these risks are unnecessary, they should be avoided [23]. Therefore, COVID-19 infection does not affect pregnant women’s request for cesarean section.

In this study, pregnant women in provincial hospital were more likely to suffer from anxiety disorder, depression and insomnia. Life and job pressures in the provincial capital city are relatively intense. The majority of pregnant women will insist on reporting to work until a few days before giving birth. Pregnant women are kept occupied by heavy workloads, domestic tasks and child care. Therefore, it is crucial for families, healthcare organisations, and employers to support and assist pregnant women throughout their pregnancy. They ought to share the burden of pregnant women, provide them with adequate psychological support, and be aware of their mood swings. and refer them to the hospital if necessary. In addition, studies have shown that patients have a strong willingness to communicate with doctors, and the lack of communication will lead to disputes between doctors and patients [24].Long waiting times for medical treatment and inpatient beds, difficulty scheduling appointments, and less time to communicate with doctors are the reasons for pregnant women’s worse mental status. Provincial hospitals should focus on improving medical services, strengthening patient-oriented communication between doctors and patients, simplifying the admission procedure and shortening the waiting time so that patients can have a better medical experience.

Pregnancy-related anxiety is inversely associated to social support and family care. Strong social support and family care are protective factors against pregnancy-related anxiety and can alleviate it [25]. Previous study [26] has shown that social support can regulate the individual’s psychological stress, thereby minimising the adverse effects of stress. Because different individuals receive different forms or levels of social support, their psychological state in the face of stress varies. Medical personnel can play a crucial role in supporting and protecting pregnant women during the COVID-19 epidemic. Pregnant women can be educated about the hazards of COVID-19 during pregnancy, as well as preventive measures they can take to reduce their risk of infection. In order to assist them in coping with the pandemic-related stressors, counselling can also be provided. To limit the risk of COVID-19 exposure in medical settings, several medical professionals have resorted to providing virtual prenatal care. This can include virtual check-ins, telemedicine consultations, and remote maternal and fetal health monitoring. Medical staff can screen pregnant women for COVID-19 symptoms and risk factors during prenatal visits and, if necessary, refer them for testing. This can aid in early detection of infections and prevent transmission to other patients and medical staff. It is important for medical staff to work closely with pregnant women to develop individualized care plans that take into account their specific needs and circumstances during the COVID-19 epidemic.

As far as we are aware, this is the first study to evaluate pregnant women in different hospitals and cities during the COVID-19 outbreak in China for symptoms of anxiety, depression, and insomnia. It’s also the first study to investigate pregnant women’s willingness to terminate their pregnancy during Omicron strain epidemic. This cross-sectional survey study revealed that omicron pandemic has a significant impact on maternal physiological status, which was supported by the combination of GAD-7, PHQ-9 and ISI.

The following limitations must be noted. First, given the study’s cross-sectional design, we are limited to asserting association rather than causality. Second, this study employed a convenient sample, which typically denotes a lack of statistical power to distinguish between demographic subgroups and can introduce biases. Also, because only 401 pregnant women were sampled, there may not be much generalizability. The participants may have been the most affected or those most eager to assist because the responses were voluntary. There is potential for representation bias, which is also a general limitation of questionnaire surveys.

## Conclusion

Anxiety disorder, depression and insomnia are common among pregnant women during the outbreak of Omicron strain epidemic in China, particularly in the provincial capital city. Delaying the prenatal examination is a potential contributing factor. During this period, the community and medical professionals should provide more psychological counseling, conduct health education and offer virtual prenatal care to pregnant women. Hospitals should improve medical services, strengthen patient-oriented communication between doctors and patients, simplify the admission procedure and shorten the waiting time so that pregnant women can have a better medical experience.

### Electronic supplementary material

Below is the link to the electronic supplementary material.

Supplementary Material 1

## Data Availability

All data generated or analysed during this study are included in this published article [and its supplementary information files].

## Abbreviations

COVID-19: Coronavirus disease 2019
SARS-CoV-2: Severe acute respiratory Syndrome Coronavirus 2
GAD-7: Generalized Anxiety Disorder 7
PHQ-9: Patient Health Questionnaire-9
ISI: Insomnia Severity Index
OR: odds ratio
CI: confidence interval
NA: not applicable
